# Cognitive Mechanisms in Chronic Tinnitus: Psychological Markers of a Failure to Switch Attention

**DOI:** 10.3389/fpsyg.2016.01262

**Published:** 2016-08-24

**Authors:** Krysta J. Trevis, Neil M. McLachlan, Sarah J. Wilson

**Affiliations:** Psychological Sciences, The University of Melbourne, Melbourne, VICAustralia

**Keywords:** tinnitus, cognition, attention, depression, salience, neurocognitive networks

## Abstract

The cognitive mechanisms underpinning chronic tinnitus (CT; phantom auditory perceptions) are underexplored but may reflect a failure to switch attention away from a tinnitus sound. Here, we investigated a range of components that influence the ability to switch attention, including cognitive control, inhibition, working memory and mood, on the presence and severity of CT. Our participants with tinnitus showed significant impairments in cognitive control and inhibition as well as lower levels of emotional well-being, compared to healthy-hearing participants. Moreover, the subjective cognitive complaints of tinnitus participants correlated with their emotional well-being whereas complaints in healthy participants correlated with objective cognitive functioning. Combined, cognitive control and depressive symptoms correctly classified 67% of participants. These results demonstrate the core role of cognition in CT. They also provide the foundations for a neurocognitive account of the maintenance of tinnitus, involving impaired interactions between the neurocognitive networks underpinning attention-switching and mood.

## Introduction

Chronic tinnitus (CT), commonly described as ‘ringing in the ears,’ can have a broad impact on an individual, affecting cognition, mood, social functioning, and general well-being. The overall prevalence of tinnitus varies across studies, ranging from 5 to 43% depending on the definition and criteria used (McCormack et al., 2016), with CT typically occurring in 10–15% of the general population (Henry et al., 2005). Despite its significant impact, there remains no cure or agreement on the mechanisms underpinning the maintenance of CT.

Tinnitus is often conceptualized as the result of a failure in the process of habituation (Hallam et al., 1984). Habituation is the ability to suppress information that is not primary to our goals, or indicative of a threat to our well-being. This process is important for facilitating our ability to control our cognitive resources and switch toward information that is of primary concern at a given moment in time. Since our cognitive capacity is limited, cognitive control of attention plays an important role in prioritizing access to relevant resources (Engle and Kane, 2004; Unsworth et al., 2014). This is achieved by modulating or ‘switching’ attention toward more salient or relevant stimuli and inhibiting stimuli less salient or relevant to task goals (Gazzaley and Nobre, 2012).

### A Neurocognitive Account of Attention-Switching

Cognitive control of attention is a core aspect of our executive functions, and is thought to involve three neural networks: the cognitive control network (CCN), the salience network (SN), and the affective network (AN; **Table 1**). Cognitive control of attention can be conceptualized as arising from the flexible balance of interactions between self-directed (the CCN) and sensory-directed (the SN) neural networks to determine the information that crosses an individual’s awareness threshold for further processing (Corbetta and Shulman, 2002; Menon and Uddin, 2010; Cocchi et al., 2013). This may be modulated by the emotional associations of a given stimulus (AN), resulting in increased monitoring of emotional information through increased SN–AN connectivity, or reduced top–down regulation from the CCN (Ochsner and Gross, 2005). In other words, the emotions associated with stimuli are important to consider, particularly with regard to engagement of the SN due to its high connectivity with the CCN and limbic system structures and proposed role in facilitating network de/activation (Seeley et al., 2007; Craig, 2009; Bonnelle et al., 2012).

**Table 1:** Neural networks proposed to underpin attention-switching.

Network	Function	Core structures
CCN	Goal directed orientation of cognitive, attention, and memory resources	Prefrontal cortex, intraparietal sulcus^1^
SN	Identification of relevant sensory inputs and facilitation of further processing	Dorsal anterior cingulate cortex, insula^2^
AN	Regulates the experience of emotions	Cingulate cortex, prefrontal cortex, amygdala, nucleus accumbens^3^

*CCN, cognitive control network; SN, salience network; AN, affective network; ^*1*^Corbetta and Shulman (2002), ^*2*^Menon and Uddin (2010), ^*3*^Ochsner and Gross (2005).*

**FIGURE 1:**
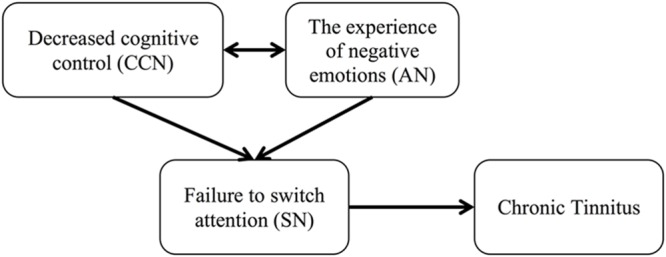
**Flow diagram of the proposed relationship between attention-switching and chronic tinnitus (CT), resulting from decreased cognitive control and emotional down regulation**.

The ability to switch our attention may be influenced by (1) our ability to control our cognitive resources, drawing on processes of inhibitory control and working memory to support flexible cognitive processing of incoming stimuli, and (2) our ability to down regulate our emotions in response to these stimuli. Specifically, proficient cognitive control enables effective attention-switching by utilizing working memory resources to maintain awareness of relevant information, and inhibitory control to resist distraction from irrelevant stimuli or thoughts (Diamond, 2013). Proficient emotion regulation influences our ability to switch attention by limiting the potential intrusion of goal-irrelevant emotionally salient information via top–down modulation of the emotions associated with a given stimulus (Ochsner and Gross, 2005; Buhle et al., 2014). In the case of tinnitus, a failure to switch attention resulting from impaired cognitive control and poor emotional down regulation may lead to ongoing engagement with the tinnitus sound, thus maintaining awareness of the sound (**Figure 1**).

### Cognitive Functioning in Chronic Tinnitus

The literature on unipolar depression has identified CCN dysfunction, notably hypoactivation, as potentially underpinning symptoms of rumination, poor attention-switching, and emotion dysregulation (Rayner et al., 2016). Behavioral indicators of these symptoms include (1) biased information processing and poor disengagement from negative stimuli, and (2) difficulties with cognitive flexibility that can be detected when assessing attention, working memory, and inhibitory control (Gotlib and Joormann, 2010). People with CT have shown impaired performance on tasks assessing these behavioral markers of CCN dysfunction when compared to people without CT (Andersson et al., 2007; Stevens et al., 2007; Pierce et al., 2012).

In particular, highly distressed individuals with tinnitus have reported greater personal salience of emotive tinnitus sentences than low-distressed and tinnitus-free individuals, which was associated with increased activation of hubs of the CCN and SN, including the insula, prefrontal cortex, and cingulate cortex (Golm et al., 2013). In addition, research investigating the cognitive functioning of people with and without tinnitus in groups matched for emotional well-being (anxiety and depressive symptoms) show impaired performance of the tinnitus group on tasks relying on cognitive control processes (Heeren et al., 2014; Jackson et al., 2014). Such findings suggest CT is associated with cognitive deficits, independent of any emotional effects that may impair cognitive function.

A recent systematic review of nine studies of working memory and attention abilities in people with tinnitus investigated subcomponents of attention, such as sustained, alerting, selective and executive attention. This showed preliminary evidence for impaired executive attention, including inhibition and switching, whereas, findings for other subcomponents were mixed (Mohamad et al., 2016). Supporting this, people with CT subjectively report a greater number of attention and memory failures in everyday life compared to hearing impaired and healthy-hearing individuals (Hallam et al., 2004; Alam et al., 2012).

These results support the hypothesis that there may be an overarching failure of cognitive control processes in people with CT, reflected in a reduced ability to switch attention away from the tinnitus sound to achieve efficient cognitive performance. This, in part, may be due to the heightened salience of the tinnitus, and its association with negative emotions, particularly anxiety (Pattyn et al., 2016) and depression (Langguth et al., 2011). Stated another way, an imbalance in the regulation of the interactions between the CCN, SN, and AN may contribute to the maintenance of tinnitus by continued assignment of attentional resources and emotional salience to the tinnitus sound (**Figure 1**). As such, furthering our understanding of cognitive control, and how this relates to the emotional well-being and subjective cognitive experiences of individuals with CT represents an important next step in understanding the role of cognition in the maintenance of CT.

The aim of the current study was to investigate cognitive markers of the ability to switch attention in people with CT to determine the role of impaired cognitive control and emotional down regulation in the maintenance of tinnitus. We hypothesized that (1) participants with CT would have slower reaction times on a cognitive control task compared to healthy-hearing participants, reflecting less efficient CCN functioning; (2) participants with CT would report poorer emotional well-being than healthy-hearing participants, reflecting the emotional impact and salience of the tinnitus; (3) both poor cognitive control and emotional well-being would predict the experience of tinnitus, reflecting their dual contribution to the maintenance of CT; and (4) subjective report of cognitive difficulties would be associated with objective cognitive task performance.

## Materials and Methods

### Participants

We recruited 26 people with self-identified CT who formed the CT group, and 29 healthy-hearing controls (HC) between 18 and 60 years using advertisements in local online newsletters and on noticeboards. Using G*Power (v3.1.9.2), a minimum total sample size of 42 was estimated to detect a moderate effect size (*f* = 0.39, derived from Heeren et al., 2014) in a repeated measures ANOVA, with power of 0.80 and α = 0.05. Inclusion criteria for the CT group were (1) the experience of tinnitus for ≥3 months, and (2) experiencing the tinnitus as constantly (always) present (Hoffman and Reed, 2004). For the HC group, individuals were screened for tinnitus symptoms and hearing impairments, resulting in the exclusion of three individuals. The study was approved by the Human Research Ethics Committee at The University of Melbourne, and all participants gave written informed consent in accordance with the Declaration of Helsinki. There were no significant differences between the two groups for age, gender or education level (all *p >* 0.10). Demographic characteristics of the two groups are summarized in **Table 2**.

**Table 2:** Participant characteristics.

	Chronic tinnitus group (*n* = 26)	Healthy control group (*n* = 26)
Mean age, years (*SD*)	40.31 (14.67)	34.15 (11.55)
Gender	42% female	54% female
Education level	77% tertiary	92% tertiary

**FIGURE 2:**
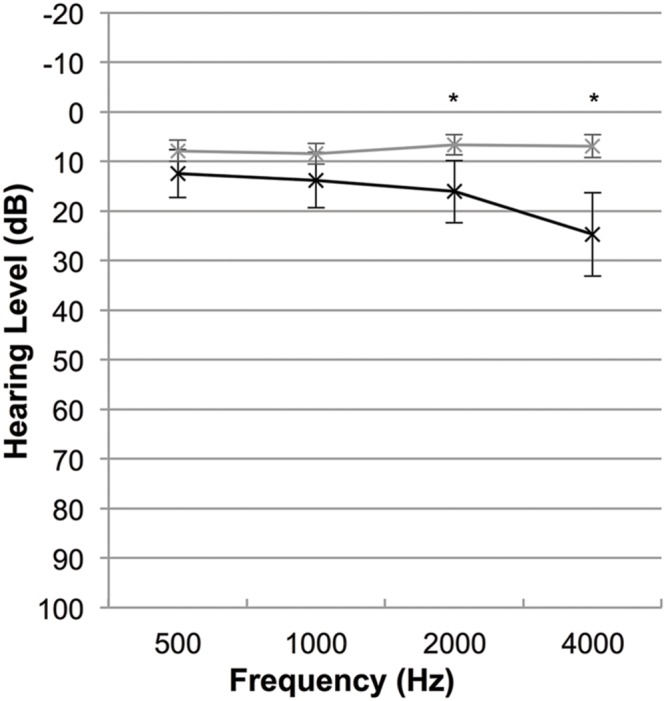
Mean audiograms for the chronic tinnitus (CT; black line) and healthy control (HC; gray line) groups (*p < 0.05). 25 dB is considered the impairment threshold.

We used the World Health Organisation’s definition of hearing impairment to determine the degree of hearing health for all participants for whom an audiogram was available (CT *n* = 25, HC *n* = 26; **Figure 2**; Concha-Barrientos et al., 2004). For each participant we calculated average hearing thresholds (dB) across four main frequencies (500, 1000, 2000, 4000 Hz) for each ear. The resulting average hearing threshold for the better ear was then classified using the World Health Organisation’s hearing impairment grading system to classify the potential impact of hearing loss on daily life. This scale ranges from no impairment (≤25 dB) to profound impairment (≥81 dB; Concha-Barrientos et al., 2004). We found no significant difference between the two groups with regard to hearing impairment [χ^2^(2, *n* = 51), 3.32, *p* = 0.19] and the average hearing threshold for each frequency was <25 dB in both groups, suggesting normal hearing ability at the group-level at each frequency. However, Mann–Whitney *U*-test comparing the groups at each frequency indicated that hearing thresholds in the CT group were significantly lower than the HC group at 2000 Hz (*U* = 212.50, *p* = 0.03) and 4000 Hz (*U* = 155.50, *p* < 0.001). Thus, we examined the data individually and found that all HC participants had normal hearing, while three participants in the CT group (12%) had impaired hearing (one slight impairment, two moderate impairment). Since removal of these three participants from the main analyses of the study did not change the significance of the results, all participants were retained for sample completeness and to reflect the heterogeneity of the presentation of CT. The tinnitus characteristics of the CT group are summarized in **Table 3**.

**Table 3:** Tinnitus characteristics (*n* = 26).

Mean years with tinnitus (SD)	13.50 (14.08)
Mean tinnitus awareness, range 0–100 (SD)	39.35 (26.45)
Mean tinnitus annoyance, range 0–100 (SD)	17.17 (21.57)
Mean tinnitus loudness, range 0–100 (SD)	41.92 (22.18)
Tinnitus laterality	
Left ear	2 (8%)
Both ears, worse in left	2 (8%)
Both ears/inside the head	14 (53%)
Both ears, worse in right	5 (19%)
Right ear	3 (12%)
Onset	
Sudden	7 (27%)
Gradual	18 (69%)
Unknown	1 (4%)
Believed cause	
Known^a^	17 (65%)
Unknown	9 (35%)

*^*a*^65% of individuals reported known causes of their tinnitus including stress (25%), music (25%), medical causes (20%), loud sound blast (10%), prolonged noise exposure (10%), head injury (5%), and hearing changes (5%).*

### Cognitive Tasks

The cognitive control and inhibition tasks were programmed using Presentation software (Neurobehavioral Systems Inc, 2014) and delivered on a 13′′ laptop screen, with responses made using a wireless mouse. The stimuli were capital letters in white Tahoma 24 point font on a black background, presented centrally on the screen. Participants were tested in a sound proof booth, and sat at a desk at a comfortable viewing distance and angle from the screen.

#### Cognitive Control

We utilized an established cognitive control task also known to activate the CCN in neuroimaging work; the ‘*n*-back’ task (Owen et al., 2005; Niendam et al., 2012). We measured cognitive control in conditions of low and high cognitive load as reaction times on this task are sensitive to manipulations of cognitive load (Braver et al., 1997; Jaeggi et al., 2007). One of eight capital letters (“C”, “G”, “H”, “K”, “P”, “Q”, “T”, or “W”) was shown on the laptop screen in a pseudorandom order for 500 ms, followed by a blank screen for 2500 ms. Participants were asked to respond by clicking the mouse whenever a target letter was shown. In the low cognitive load condition (0-back), the target was a specific letter that participants were instructed to respond to at the start of each block (e.g., “C”). In the high cognitive load condition (2-back), participants were instructed to respond to the target which occurred when a letter was the same as the one seen two previously. Each block lasted approximately 1 min and comprised 20 letters, of which 30% were targets. Each participant completed one run of the task, comprising three blocks of the 0-back and three blocks of the 2-back, presented in random order. Before each block, participants were reminded of the instructions, and could take a break if required. We also used a short 10-letter practice block to train participants on the task, for which 60% of targets had to be correctly identified in both the 0-back and 2-back conditions prior to commencing the experimental run.

#### Inhibitory Control

We used the gold-standard Stop-Signal Task to assess the inhibitory control of attention given this contributes to performance on the n-back task (Logan et al., 1997; Avila and Parcet, 2001). Participants were required to respond as quickly as possible to the letters ‘A’ and ‘Z’ when they appeared on the laptop screen using the left (‘A’) and right (‘Z’) mouse buttons respectively. The letters were preceded by a fixation cross (500 ms) and displayed for 1000 ms. Participants were instructed to inhibit their response if a letter ‘X’ was also shown on the screen (the ‘stop signal’). The ‘stop signal’ was presented in red font for 150 ms, with a delay between the onset of the primary letter (‘A’ or ‘Z’) and the stop signal of 250 ms. This delay varied by ±50 ms based on the participant’s stopping accuracy (with a minimum delay of 50 ms and a maximum of 500 ms). There was a 1000 ms break between each trial, with 25% stop trials randomly presented over a total of 160 trials (80 for each letter), presented in random order. A rest break was provided half-way through the task. Before completing the task, participants were required to achieve a minimum of 50% correct responses on the practice trials, with a minimum of 10 practice trials shown.

#### Working Memory

We used the digit-span subtest of the Wechsler Adult Intelligence Scale – Fourth edition (Wechsler, 2008) to assess working memory given its contribution to cognitive control task performance. For this test, participants were asked to repeat back strings of numbers that gradually increased in length. All three subtests of the digit-span test were administered according to the test manual. In the first subtest, Digit Span Forward, participants repeated the numbers in the same order. In the second subtest, Digit Span Backward, participants were asked to recall the numbers in the backward order. Finally in the third subtest, Digit Span Sequencing, participants recalled the numbers in ascending order (from low to high). Each correct response is awarded one point, with a maximum of 16 points for each subtest. The total score for the three subtests formed the overall digit-span score. All raw scores on this test were converted to age-scaled scores, estimated using the norms in the WAIS-IV manual (Wechsler, 2008).

### Emotional Well-being Measures

We used established questionnaires with well-documented psychometric properties as indicated by their internal consistency scores (α) to assess anxiety-proneness and depressive symptoms. The trait subscale of the State Trait Anxiety Inventory (STAI; Spielberger et al., 1983) was used to assess anxiety-proneness (α = 0.91) and depressive symptoms were assessed using the Beck Depression Inventory (BDI-II, α = 0.93; Beck et al., 1996).

### Procedure

After providing informed consent, participants underwent a hearing test and provided sociodemographic information. The CT group also provided a history of their tinnitus experiences using the Tinnitus Case Sample History Questionnaire (Langguth et al., 2007), and we assessed the subjective impact of their tinnitus on daily life using the Tinnitus Handicap Inventory (THI; Newman et al., 1996). All participants also completed the Cognitive Failures Questionnaire (CFQ) to assess the subjective experience of memory and attention failures (Broadbent et al., 1982). Participants then completed the working memory and inhibition tasks, presented in counter-balanced order, after which they completed the cognitive control task in conditions of silence and in the presence of a repetitive background noise (control condition), with the order counterbalanced. Participants finished with the emotional well-being questionnaires.

### Data Analysis

The Statistical Package for the Social Sciences Version 22 (SPSS) was used for all analyses. We tested the data for assumptions of parametric testing and identified extreme outliers, for which we applied a 90% winsorisation (<1% of data; Field, 2008). Where the assumptions of parametric tests were not upheld, more conservative non-parametric tests and log transformations for reaction time data were used to confirm the results. As no differences between parametric and non-parametric test outcomes were observed, the results of parametric tests on the raw data are reported here. We calculated effect size estimates for all statistical tests using eta squared (η^2^) for analysis of variance models (ANOVA) and Cohen’s (*d)* for independent sample *t*-test (Fritz et al., 2012).

To test hypothesis 1, that the CT group would have slower mean reaction times on the cognitive control task compared to the HC group, we performed an ANOVA on the reaction time data, with group as the between-subjects factor and cognitive load (0-back, 2-back) as the within-subjects factor. For the inhibitory control task, individual performance was first estimated using the integration method to calculate each participant’s mean stop signal reaction time (SSRT; Verbruggen et al., 2013). We then compared the average performance of the CT and HC groups using an independent samples *t*-test. For the working memory task, we compared the Digit Span scaled scores for the CT and HC groups using independent samples *t*-test for the total test score and for each subtest. As inhibitory control and working memory are component skills of the n-back task, we performed secondary level analyses to adjust for these on the cognitive control task using analysis of covariance (ANCOVA) models.

To test hypothesis 2, that the CT group would report poorer emotional well-being than the HC group, we performed independent samples *t*-test on the group mean scores of the BDI-II and STAI-T measures. As mood and anxiety can influence cognitive performance, we also performed secondary level analyses to adjust for these on the cognitive control task, again using ANCOVA models.

To test hypothesis 3, that poor cognitive control and emotional well-being would independently predict the experience of tinnitus (i.e., group membership) we conducted a stepwise discriminant function analysis (DFA) with α = 0.05 as the criterion for variables entering the analysis. We identified relevant cognitive and emotional well-being variables from those showing significant group differences in the analyses described above, and then examined their effectiveness in differentiating people with and without CT.

Finally, to test hypothesis 4, that subjective report of cognitive difficulties would be associated with objective cognitive task performance, we used one-tailed Pearson correlations (*r*) to determine if measures of objective cognitive ability and emotional well-being were significantly correlated with participant scores on the CFQ.

## Results

### Decreased Cognitive Control in Chronic Tinnitus

**FIGURE 3:**
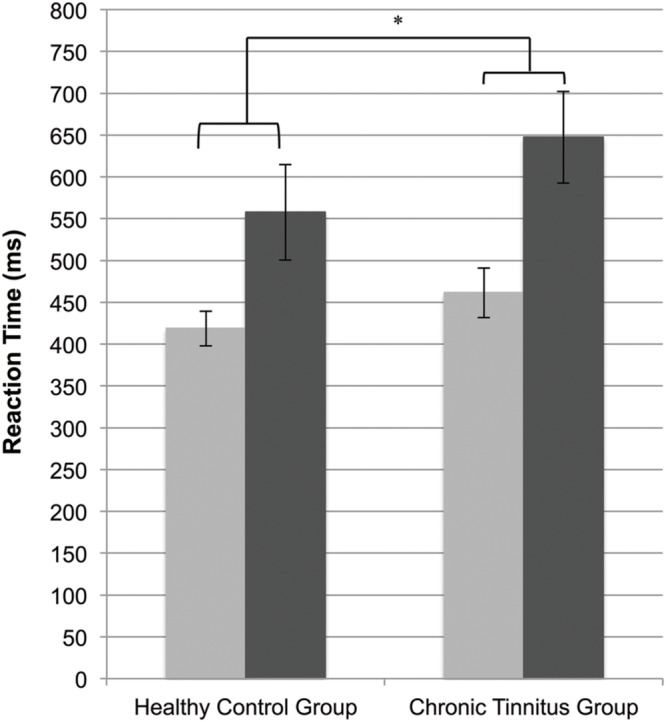
Mean reaction times for the chronic tinnitus (CT) and healthy control (HC) groups on the 0-back (light gray) and 2-back (dark gray) versions of the cognitive control task (*p < 0.05).

In support of our first hypothesis there was a significant main effect of group, indicating that individuals with tinnitus responded more slowly on the 2-back and 0-back conditions of the cognitive control task compared to the HC group in silence [*F*(1,50) = 7.23, *p* = 0.010, η^2^ = 0.13; see **Figure 3**]. As expected, there was also a main effect of cognitive load [*F*(1,50) = 92.33, *p* < 0.001, η^2^ = 0.64], with faster reaction times in the 0-back condition (*M* = 440.12, *SE* = 8.79, 95%CI 422.46, 457.77) compared to the 2-back condition (*M* = 602.53, *SE* = 19.21, 95%CI 563.95, 641.11). There was no interaction between cognitive load and group [*F*(1,50) = 1.95, *p* = 0.169, η^2^ = 0.01].

In addition to group differences on the cognitive control task, the CT group were also significantly slower on the inhibitory control task (*M* = 242.01, *SD* = 47.25) than the HC group [*M* = 217.31, *SD* = 35.80, *t*(50) = 2.13, *p* = 0.039, *d* = 0.59]. In contrast, there were no significant working memory differences between the CT (*M* = 11.23, *SD* = 3.14) and HC groups [*M* = 11.65, *SD* = 3.17, *t*(50) = -0.48, *p* > 0.250, *d* = 0.13] on the Digit Span total or subtest scores (all *p* > 0.250).

When we adjusted cognitive control scores for performance on the inhibitory control task by entering SSRT as a covariate, the main effect for group remained, although exhibited a smaller effect size and marginal significance [*F*(1,49) = 3.96, *p* = 0.052, η^2^ = 0.07]. This suggests cognitive control task performance partially reflects inhibitory control performance. When we adjusted scores for working memory ability by entering the Digit Span total test score as a covariate, the main effect for group remained significant and showed a similar effect size [*F*(1,49) = 6.85, *p* = 0.012, η^2^ = 0.12]. While the groups did not differ significantly for demographic or hearing abilities, these factors could influence cognitive control and as such we assessed each factor as a potential covariate to determine the robustness of our findings. In support of our results, the main effect of group remained significant after accounting for age [*F*(1,49) = 4.33, *p* = 0.043, η^2^ = 0.07], hearing ability [*F*(1,48) = 7.32, *p* = 0.009, η^2^ = 0.13], gender [*F*(1,49) = 6.64, *p* = 0.013, η^2^ = 0.12], and education [*F*(1,49) = 6.26, *p* = 0.016, η^2^ = 0.12].

We also assessed if reduced cognitive control was associated with CT factors, including (a) the subjective loudness of the tinnitus sound, (b) the perceived impact of tinnitus on daily life, and (c) any perceived worsening of the tinnitus during task performance. Spearman correlations indicated that neither subjective loudness nor tinnitus handicap were associated with performance of any of the cognitive tasks (all *p* > 0.10). Similarly, independent *t*-test showed no significant differences for participants who experienced a worsening of their tinnitus (*n* = 12) compared to no change (*n* = 14) whilst performing the tasks (all *p* > 0.10).

**FIGURE 4:**
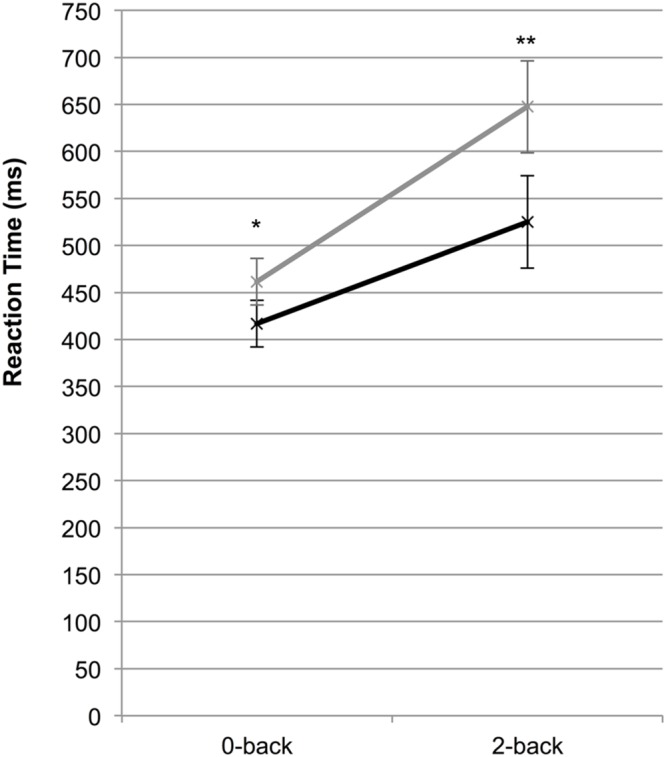
Mean reaction time for the chronic tinnitus group (CT; gray line) in silence (i.e., tinnitus sound present) and the healthy control group (HC; black line) in the presence of a repetitive background sound while performing the cognitive control task (*p < 0.05, **p < 0.001).

Finally, we assessed whether our main effects could be replicated in the presence of background sound and found a significant main effect for group [*F*(1,50) = 6.51, *p* = 0.014, η^2^ = 0.12] and cognitive load [*F*(1,50) = 83.74, *p* < 0.001, η^2^ = 0.63], as well as an interaction effect where reaction times increased more for the CT group between the 0- and 2-back conditions [*F*(1,50) = 4.23, *p* = 0.045, η^2^ = 0.08]. To distinguish whether these results were due to the presence of the tinnitus sound *per se* or a failure to switch attention away from the sound in the CT group, we compared the CT group’s task performance in silence (tinnitus sound present) to the HC group’s performance in the presence of the repetitive background sound. Here, we found a larger interaction effect [*F*(1,50) = 5.74, *p* = 0.020, η^2^ = 0.10] indicating that when a noise source is present (tinnitus or background sound), there is a greater performance cost for the CT group as cognitive load increases (**Figure 4**). Consistent with the earlier analyses, we also observed main effects for group [*F*(1,50) = 13.38, *p* = 0.001, η^2^ = 0.21] and cognitive load [*F*(1,50) = 85.27, *p* < 0.001, η^2^ = 0.63].

### Poor Emotional Well-being in Chronic Tinnitus

In support of our second hypothesis, the CT group reported significantly more symptoms of depression (*M* = 11.79, *SD* = 10.03) than the HC group (*M* = 6.38, *SD* = 5.78); *t*(39.96) = 2.38, *p* = 0.022, *d* = 0.68. In the CT group, 43% of people reported depressive symptoms in the mild-severe range (23% mild, 12% moderate, 8% severe) compared to 12% of the HC group (8% mild, 4% moderate, 0% severe). One tinnitus participant had a current diagnosis of clinical depression and was identified as a statistical outlier, however, removal of this participant did not change the significance of any of the described effects, and hence she was retained for sample completeness. No differences were observed between groups for the trait of anxiety-proneness *t*(50) = 1.35, *p* = 0.182, *d* = 0.37. We also assessed the influence of emotional well-being on performance of the cognitive control task, first by entering BDI-II scores as a covariate in the analysis. This indicated that the main effect of group remained significant with a similar effect size [*F*(1,49) = 7.54, *p* = 0.008, η^2^ = 0.13]. Similarly, when we entered anxiety-proneness as a covariate the groups remained significantly different, again with similar effect size [*F*(1,49) = 8.50, *p* = 0.005, η^2^ = 0.14].

### Predictors of Chronic Tinnitus

For factors that showed group differences (cognitive control, inhibitory control, and depressive symptoms), we conducted a DFA to identify those factors that were most significant in discriminating between people with CT and healthy controls. Assumption checks for the stepwise DFA showed moderately strong correlations between the 0-back and 2-back conditions of the cognitive control task for both groups [*r_s_*(CT) = 0.40, *p* = 0.022; *r_s_*(HC) = 0.40, *p* = 0.025]. As such, to address the assumption of multicollinearity we removed the 0-back condition from the DFA in favor of the more cognitively demanding 2-back task. In this model, 2-back performance was identified as the core discriminating factor of people with and without CT, Wilk’s λ = 0.90, *F*(1,50) = 5.47, *p* = 0.023, with the addition of depressive symptoms in the second step further improving the model, Wilk’s λ = 0.81, χ^2^(2) = 10.12, *p* = 0.006. Combined, these two factors correctly classified 67% of cases and showed good sensitivity for participants with CT (65%), indicating that poor cognitive control and greater depressive symptoms were predictive of CT group membership. There was also good specificity (69%) for the HC group, suggesting low depressive symptoms and more effective cognitive control were consistent features of this group. Inhibitory control did not significantly contribute to the model.

To explore the data for effects relating to tinnitus severity, we conducted Spearman correlation (*r_s_*) analyses between severity of tinnitus impact, cognitive control performance, and depressive symptoms. Since severity of the impact is considered a separate factor from the constant presence of the sound in the ‘vicious cycle’ of CT (Jastreboff et al., 1996), this analysis aimed to identify if either factor relating to its presence, also related to its perceived severity. We found that the degree of tinnitus impact was selectively associated with depressive symptoms (*r_s_* = 0.42, *p* = 0.033), but not with cognitive control (*r_s_* = -0.32, *p* = 0.112) despite both factors playing a role in correctly classifying people with and without CT. Combined these findings suggest ongoing awareness of the tinnitus sound may relate to reduced cognitive control, whereas depressive symptoms may determine the severity of its impact on an individual’s daily life.

### Correlates of Subjective Cognitive Complaints

The CT group reported significantly more subjective cognitive complaints (*M* = 43.07, *SD* = 12.00) than the HC group (*M* = 34.35, *SD* = 14.51); *t*(50) = 2.36, *p* = 0.022, *d* = 0.66, consistent with their objective performance on the cognitive control and inhibition tasks. Interestingly, we found differences in the pattern of associations between the CT and HC groups. The HC group showed an association between objective cognitive performance and subjective cognitive complaints, with significant correlations between scores on the CFQ and 2-back performance (*r* = 0.38, *p* = 0.028), between CFQ and SSRT scores (*r* = 0.57, *p* = 0.001), but no significant associations between CFQ scores and depressive symptoms or anxiety-proneness. In contrast, the CT group showed no significant associations between objective cognitive performance and subjective cognitive complaints. Rather, CFQ scores were associated with anxiety-proneness (*r* = 0.69, *p* < 0.001) and with depressive symptoms (*r* = 0.55, *p* = 0.002).

## Discussion

The present study addresses the role of attention-switching in CT by investigating two key aspects of attention-switching hypothesized to show impairments in this population, namely cognitive control and the experience of negative emotions. Consistent with our first hypothesis, we demonstrated slower reaction times in cognitive and inhibitory control in people with CT, whereas working memory function was similar to healthy controls. Consistent with our second hypothesis, we also found poorer emotional well-being in people with CT reflected by elevated depressive symptoms, whereas trait anxiety (anxiety-proneness) was similar to healthy controls. Third, we demonstrated a core role of reduced cognitive control in accurately classifying people with CT, while depressive symptoms were associated with its perceived severity. Taken together, these findings suggest that reduced control of the ability to switch attention may constitute a cognitive mechanism that maintains the awareness and severity of the tinnitus sound.

### Cognitive Control and Tinnitus Awareness

Our results indicate that people with CT are less proficient at performing a cognitive control task compared to people with healthy-hearing, an effect which remained after adjusting for (1) performance on tasks assessing the component skills of inhibitory control and working memory, and (2) emotional well-being, including depressive symptoms and anxiety-proneness. Importantly, all participants were able to accurately perform and execute the cognitive tasks (*n*-back, stop-signal, and digit-span) and the groups were similar in age and hearing ability, two factors thought to influence cognitive abilities (Baltes and Lindenberger, 1997). Therefore, given the similarities in demographic variables between the groups, and the robustness of the effect after accounting for other cognitive, emotional and behavioral factors that could influence the results, it appears likely that the observed impairment in cognitive control is primarily related to the presence of CT.

An additional investigation of task performance in the presence of repetitive background sound provided further support for a failure to switch attention underpinning the observed impairment in cognitive control. In particular, replication of poorer performance of the CT group on the cognitive control task compared to the HC group in the presence of background noise suggests that the results were not due to the presence of the constant tinnitus sound itself, but rather a failure in attention-switching in people with CT. Specifically, there was a greater performance cost for people with CT as cognitive load increased when both groups were exposed to potential auditory distractors, suggesting that impaired top–down regulation of less relevant sensory information may underpin maintained awareness of the tinnitus sound. Consistent with this interpretation, we found no evidence that cognitive performance was influenced by factors relating to the tinnitus sound itself, including its volume, impact, or perceived worsening during task performance.

Since we found no overall differences in hearing ability between the groups, nor any influence of hearing ability on cognitive performance, and our main findings did not change with removal of participants with hearing impairment in the CT group, we feel it is unlikely that hearing ability accounts for the present results. However, given previous research has shown an association between uncorrected hearing impairment and cognitive functioning, particularly in older adults (Wayne and Johnsrude, 2015), the inclusion of individuals with hearing impairments may represent a limitation of our study. Future research directly investigating the relationships between cognition, age, and hearing ability may help to clarify the potential influence of these factors on the experience of CT, including the point at which hearing loss may pose a significant risk to cognition.

The similarity between groups for working memory may reflect this task’s reliance on performance accuracy over processing speed, the latter being crucial to the performance of the cognitive control and inhibition tasks. This suggests that tasks relying on information processing speed may provide more sensitive measures of attention-switching in people with CT than measures of accuracy alone, potentially accounting for discrepant findings in previous research (Mohamad et al., 2016).

### Psychological Salience and Tinnitus Severity

We have shown that there is a subjective emotional cost associated with the severity of tinnitus. Depressive symptoms not only predicted the presence of tinnitus but were also correlated with its severity, suggesting they play a specific role in determining the psychosocial impact of CT. This is consistent with recent findings of a core role of depressive symptoms in maintaining the awareness and impact of CT (Trevis et al., 2016). Depressive symptoms were also correlated with perceived cognitive difficulties in everyday life in the CT group, which may reflect the presence of a negative cognitive bias, which is a characteristic feature of depression (Rayner et al., 2016). These findings emphasize the importance of considering the psychological well-being of people with CT with regard to both effective treatment, and furthering our understanding of how cognition and emotion regulation, particularly with regard to low mood, can interact to maintain tinnitus awareness and severity. In addition, assessing the cognitive functioning of people with CT with or without depression would help delineate the contributions of mood and cognitive control to the presence and severity of CT.

### Chronic Tinnitus and Attention-Switching

The present results indicate that both cognitive control and emotional regulation, two processes proposed to influence the ability to switch attention, are impaired in people with CT. Importantly, our results also suggest these processes serve different functions that are consistent with research on chronic pain, a condition considered to have similar underlying mechanisms to CT (Moller, 2007). In particular, our results suggest that depressive symptoms, cognitive control, and to some degree inhibitory control, may relate to the ongoing perception of the tinnitus sound, while emotion regulation, specifically of depressive symptoms, may relate to the perceived severity of its impact. Of note, symptoms of depression appear to be involved in maintaining both the awareness and severity of CT, which may reflect distinct roles for the cognitive and affective features of depression in specific aspects of tinnitus maintenance. For example, cognitive depressive symptoms (associated with CCN dysfunction) may be involved in the ongoing awareness of the sound while affective or somatic depressive symptoms (associated with AMN/AN dysfunction) may increase the severity and psychological salience of the tinnitus sound. In the chronic pain literature, research suggests that attention factors selectively modulate the awareness of pain, while mood selectively modulates the unpleasantness of the pain (Bushnell et al., 2013). Impaired cognitive control has also been implicated in anxiety (Derryberry and Reed, 2002) and depression (Fales et al., 2008), with both psychological conditions having a high incidence rate in CT populations (Zirke et al., 2013). As such, we propose that the ongoing awareness and severity of CT is underpinned by a failure of top–down cognitive resources, specifically cognitive and emotional control, resulting in a reduced ability to switch attention away from the tinnitus sound.

### A Neurocognitive Approach to Chronic Tinnitus

A strength of our study involved the use of the *n*-back task, which is well-established in the cognitive neuroimaging literature to activate the CCN. In light of this, our findings suggest that people with CT may show hypoactivation of the CCN, decreasing top–down inhibition of the tinnitus sound. This, in turn, would increase the psychological salience of the sound (SN), which, in combination with reduced top–down inhibition of the AN by the CCN, may facilitate negative thinking about the sound (e.g., rumination) and emotion dysregulation (e.g., low mood).

In addition, the CCN is antithetical to the autobiographical memory network (AMN), also known as the ‘resting state’ or ‘default mode’ network, which is associated with introspection and rumination (Sheline et al., 2009; Rayner et al., 2016). To engage with the outside world through goal-directed behaviors we need to reduce self-focused thinking by suppressing the AMN, allowing the CCN to direct our cognitive resources and actions (Rayner et al., 2016). In CT, hypoactivation of the CCN may be related to a hyperactive, internally focused AMN, as suggested by resting-state connectivity data showing a highly connected auditory-limbic resting state network in people with tinnitus compared to people without tinnitus (Maudoux et al., 2012).

**FIGURE 5:**
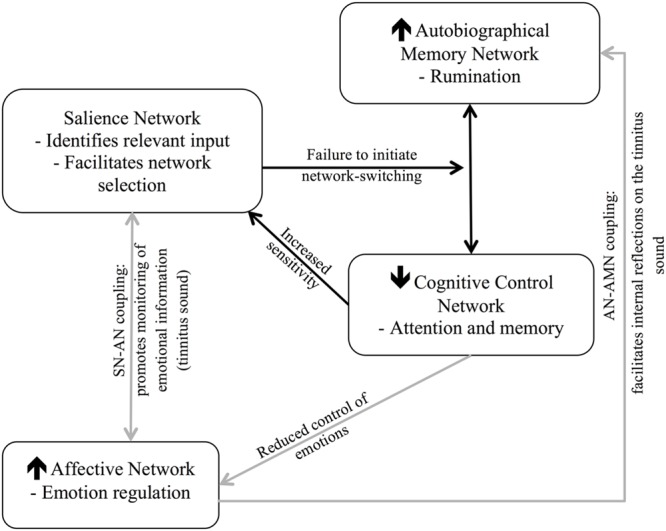
**Neurocognitive networks proposed to maintain tinnitus awareness (black arrows) and tinnitus severity (gray arrows)**.

As illustrated in **Figure 5**, this neurocognitive account conceives tinnitus as a functional imbalance in the interaction of neurocognitive networks. Importantly, it distinguishes the neural networks associated with maintained awareness of the tinnitus sound, underpinned by a hypoactive CCN and hyperactive AMN, which facilitates ongoing attention toward a salient tinnitus sound. It also distinguishes the severity of the impact of tinnitus, underpinned by dysfunction of the AN, AMN, and SN, facilitating negative, internally focused processing of the sound. Considered this way, CT may reflect a fundamental failure of the CCN to inhibit the tinnitus percept when it is emotionally salient to an individual.

In support of the neurocognitive model, auditory processing of aversive sounds has shown reliable activation in the affective and SNs, particularly in the amygdala which is a core AN hub with strong connections to auditory processing (Zald and Pardo, 2002; Kumar et al., 2012). In addition, rapid habituation of the amygdala to aversive auditory stimuli has been proposed to be mediated by the SN (Büchel et al., 1998). There is also evidence of top–down control of auditory processing and associated neuroplasticity of auditory regions via CCN and SN hubs, including pre-frontal and parietal cortices, insula and the anterior cingulate cortex (Tzourio et al., 1997; Westerhausen et al., 2010). As such, there are established links between the functioning and plasticity of auditory pathways and the neurocognitive networks associated with cognitive control, emotion regulation and the salience of incoming sensory information (Westerhausen et al., 2010; Moore, 2013; Moreno and Bidelman, 2014). In light of this, the application of neuroimaging techniques to assess the integrity of these networks in people with and without CT would be beneficial to further assess the potential role of neurocognitive mechanisms in CT.

These network interactions are exemplified in a pilot real-time fMRI auditory control training study which found that conscious suppression of auditory cortex activation was associated with greater activation of the CCN and deactivation of the antithetical AMN, supporting the proposed role for these large-scale neurocognitive networks in the maintenance of CT (Haller et al., 2009). Consistent with this, a range of auditory-based activities have been shown to be protective of the psychological impact of CT, including auditory training therapies, real-time fMRI training of auditory control, and music training (Flor et al., 2004; Haller et al., 2009; Schneider et al., 2009). In particular, music training may be effective in alleviating tinnitus symptoms due to its effectiveness in improving (1) emotion regulation through its effect on the AN (Moore, 2013), and (2) cognitive functions via ‘far transfer’ effects on attention and working memory processes, which are mediated by CCN functions (Strait et al., 2010; Argstatter et al., 2012; Moreno and Bidelman, 2014). Investigating the possibility of training attention abilities through music or auditory control tasks may be a potential mechanism to attenuate the awareness and severity of CT worthy of further research.

Furthermore, psychological treatments aimed at strengthening goal-directed attentional control, such as mindfulness and cognitive behavior therapy (CBT) may benefit people with CT by increasing the proficiency of the CCN and improving its regulation of associated networks (SN, AN). Both CBT and mindfulness have been shown to be effective in lessening the impact of CT and improving psychosocial well-being (Philippot et al., 2011; Cima et al., 2012, 2014; Kreuzer et al., 2012). Specifically, evidence of neuroplasticity in regions of the neurocognitive networks proposed to underpin the impact and presence of CT have been found following CBT or mindfulness interventions (Frewen et al., 2008; Chiesa and Serretti, 2009; Brewer et al., 2011). In addition, the functioning of core hubs in these networks, including the dorsal anterior cingulate cortex and regions of the prefrontal cortex have been found to predict the effectiveness of CBT. This suggests that examining the functioning of the CCN and SN as part of ‘treatment readiness’ prior to commencing CBT may help to improve prognosis, engagement, and potential treatment benefits (Klumpp et al., 2014).

## Conclusion

Our findings suggest that people with CT are less proficient in switching attention away from the tinnitus sound compared to people with healthy hearing. This highlights a core role of cognition, particularly cognitive control, in maintaining awareness of the tinnitus, as well as reduced emotional down regulation, with depressive symptoms associated with the severity of the tinnitus. Importantly, cognitive control and depressive symptoms provide targets for future treatment studies aimed at reducing awareness of the tinnitus sound and improving the well-being of people with CT. Finally, our results provide a foundation for establishing a new neurocognitive account of CT. This account suggests that a functional imbalance of specific large-scale neural networks associated with processes of attention-switching and emotion regulation may underpin chronic awareness of the tinnitus sound and the severity of its impact.

## Author Contributions

All authors contributed to the study concept. KT collected the data and completed the data analysis and interpretation under the supervision of SW. KT and SW wrote the manuscript, and NM provided critical revisions. All authors approved the final version of the manuscript for submission.

## Conflict of Interest Statement

The authors declare that the research was conducted in the absence of any commercial or financial relationships that could be construed as a potential conflict of interest.
